# Association between oral health status and peptic ulcer disease: evidence from a nationally representative survey in Hungary

**DOI:** 10.3389/froh.2026.1739255

**Published:** 2026-02-18

**Authors:** Battamir Ulambayar, Amr Sayed Ghanem, Attila Csaba Nagy

**Affiliations:** Department of Epidemiology, Faculty of Health Sciences, University of Debrecen, Debrecen, Hungary

**Keywords:** epidemiology, Hungary, oral health, peptic ulcer disease, periodontitis

## Abstract

**Background:**

Peptic ulcer disease (PUD) remains a significant public health issue, with established risk factors including Helicobacter pylori infection, nonsteroidal anti-inflammatory drugs (NSAID) use, and lifestyle factors. Emerging evidence suggests a link between poor oral health and systemic diseases, yet its association with PUD is understudied, particularly in Central and Eastern Europe. This study aimed to investigate the relationship between oral health status and the presence of PUD in a nationally representative Hungarian population.

**Methods:**

Using cross-sectional data from the 2,019 European Health Interview Survey in Hungary (EHIS), we analyzed oral health indicators (decayed teeth, bleeding gums, loose teeth, tooth extractions, dental restorations, and self-perceived oral health) and constructed a composite oral health status via exploratory factor analysis. Multivariable logistic regression assessed the association between oral health and self-reported PUD, adjusting for sociodemographic, lifestyle, mental health, and comorbidity factors.

**Results:**

Active dental and periodontal disease (OR = 1.54, 95% CI: 1.07–2.20, *p* = 0.017) and a history of dental and periodontal disease (OR = 2.17, 95% CI: 1.01–4.67, *p* = 0.047) were significantly associated with higher odds of self-reported PUD. Older age, female sex, smoking, depression, and selected comorbidities were also associated with PUD.

**Conclusions:**

Poor oral health, particularly indicators of active periodontal disease, was independently associated with PUD in this nationally representative cross-sectional sample. These findings suggest a potential connection between oral health status and gastrointestinal health, which warrants further investigation in longitudinal studies.

## 1 Introduction

Peptic ulcer disease (PUD) remains an important public health problem, affecting populations worldwide and contributing to substantial morbidity and impaired quality of life (1, 2). The epidemiology of ulcer disease in Europe reflects this global trend, although variation across regions persists. Although mortality from diseases of the digestive system has declined since the mid-1990s in Europe, Hungary still exhibits one of the highest rates in the WHO European Region, and digestive disorders remain among the five leading causes of death in the country (3). While the incidence of PUD has declined in recent decades with improved management of Helicobacter pylori infection and the use of proton pump inhibitors, PUD continues to pose a burden, particularly among specific sex and age groups and individuals with comorbid conditions (4). Established risk factors include H. pylori infection (5), long-term use of nonsteroidal anti-inflammatory drugs (NSAIDs) (6), smoking, alcohol consumption, and stress-related disorders (7). However, less is known about the role of other health determinants, such as oral health, in ulcer occurrence.

Oral health is increasingly recognized as an integral component of general health, with poor oral conditions linked to a wide range of systemic diseases, including cardiovascular disease (8), diabetes (9), gastrointestinal (10) and respiratory illness (11). Potential biological mechanisms include chronic inflammation, alterations of the oral microbiome, and systemic immune responses triggered by periodontal disease and tooth loss (12). Beyond biological pathways, poor oral health may also reflect broader health inequalities and limited access to preventive care, which are themselves associated with chronic disease outcomes (13).

Despite growing evidence of the systemic impact of oral health, its relationship with PUD has rarely been investigated. Noteworthy findings from Asia, particularly from Korea (14) and Japan (15), have reported modest but significant associations between self-reported oral health status and a history of PUD. However, the evidence from Central and Eastern Europe is particularly scarce. Understanding whether oral health is associated with PUD may provide new insights into disease prevention and highlight opportunities for integrating oral health into general health promotion strategies.

The present study aimed to investigate the relationship between oral health status and PUD in the Hungarian adult population, using data from the 2019 European Health Interview Survey (EHIS). By analyzing multiple indicators of oral health and constructing a composite oral health score, we aimed to determine whether poor oral health is associated with an increased likelihood of PUD, after adjusting for sociodemographic, lifestyle, mental health, and comorbidity factors.

## 2 Methods

### 2.1 Study design and data

This study used data from the 2019 EHIS conducted in Hungary, which employs a complex, multistage, stratified probability sampling design to obtain a nationally representative sample of the non-institutionalized adult population. Sampling weights provided by the Hungarian Central Statistical Office were applied to account for unequal probabilities of selection, non-response, and post-stratification by age, sex, and region. Survey strata and primary sampling units were specified in all analyses to appropriately reflect the complex survey design and ensure valid variance estimation. The survey is coordinated by Eurostat, designed to collect information on health status, health determinants, and healthcare use among European populations. The Hungarian EHIS 2019 dataset included detailed sociodemographic characteristics, lifestyle factors, comorbidities, and self-reported oral and general health indicators from 5,603 respondents (16).

Participants were eligible for inclusion if they were aged 18 years or older and had complete information on PUD status, oral health indicators, and covariates included in the multivariable models. Respondents were excluded if data were missing for PUD status, any oral health variables used in the exploratory factor analysis, or key sociodemographic, lifestyle, mental health, or comorbidity covariates. Of the 5,603 respondents in the Hungarian EHIS 2019 dataset, 431 individuals (7.7%) were excluded based on these criteria, resulting in a final analytical sample of 5,172 participants.

### 2.2 Variables

The primary outcome variable was self-reported PUD. PUD status was determined using a structured, multi-step questionnaire. Participants were first asked whether they had ever had a PUD. Those responding affirmatively were subsequently asked whether the condition had been diagnosed by a medical doctor and whether they had received any form of treatment for the condition. A PUD diagnosis was considered confirmed only when participants answered “yes” to all three questions. Oral health status was assessed using several indicators: presence of decayed teeth, filled teeth, bleeding gums, loose teeth, history of tooth extraction due to decay or loosening, presence of dental restorations (crown/bridge/denture), missing teeth without dentures, and the number of missing teeth, and self-perceived oral health (17). Using these oral health status variables, variables related to active dental and periodontal disease, as well as a history of dental and periodontal disease, were created based on the exploratory factor analysis (EFA) results.

Covariates included demographic and socioeconomic factors such as age, sex, educational attainment, household income quintiles, and self-perceived general health; lifestyle factors including smoking status, alcohol consumption, categorized based on EHIS self-reported frequency and quantity of intake as non-alcohol use, moderate use, or heavy use, and body mass index; and mental health status, assessed using the Patient Health Questionnaire-8 (PHQ-8) and categorized as no depressive symptoms, other depressive symptoms, or major depressive symptoms (18). Information on medication use, including NSAIDs, was not available in the EHIS dataset. Given the established role of NSAIDs in the pathogenesis of PUD, we included selected comorbidities, specifically cardiovascular, rheumatic, liver, and kidney diseases, as proxy indicators of potential long-term NSAID exposure or other ulcerogenic pharmacotherapy. These conditions are commonly associated with chronic use of NSAIDs, antiplatelet agents, or anticoagulants in routine clinical practice and have been used as proxies for medication exposure in previous population-based studies. However, it should be noted that this proxy-based approach may not fully capture individual-level medication use.

### 2.3 Statistical analysis

Descriptive analyses were performed to compare baseline characteristics between participants with and without PUD. Differences across categorical variables were assessed using the Chi-square test. EFA with tetrachoric correlations was conducted to validate the composite oral health status using binary indicators (19), including the presence of caries, filled teeth, bleeding gums, loose teeth, history of tooth extraction, presence of dental restorations or prosthesis, and missing teeth without dentures. Kaiser-Meyer-Olkin and Bartlett's tests assessed data suitability (20). Although the overall KMO value indicated marginal sampling adequacy, several item-level KMO values were acceptable, Bartlett's test strongly supported factorability, and the oral health indicators showed clear theoretical coherence. Variables with low factor loadings, high uniqueness, or substantial cross-loadings were excluded to improve interpretability and reduce redundancy.

Multivariable logistic regression models were applied to examine the associations between the composite oral health status, including active dental and periodontal disease and the history of dental and periodontal disease, and the presence of PUD, adjusting for demographic, socioeconomic, lifestyle, mental health, and comorbidity factors. Self-perceived general health status was excluded from the model due to multicollinearity with comorbidities, although it was significantly associated with PUD by the chi-square test. The Area Under the Receiver Operating Characteristic Curve (AUC) was calculated to assess the model's ability to discriminate effectively. Results are presented as odds ratios (OR) with 95% confidence intervals (CI). Statistical significance was set at *p* < 0.05. Potential interactions between depression, smoking, and other lifestyle factors were considered conceptually; however, formal interaction terms were not included in the final models due to limited statistical power and the exploratory nature of the analysis. All statistical analyses were conducted using survey-adjusted procedures to account for the complex sampling design of the EHIS. Sampling weights, strata, and primary sampling units were incorporated using the svy commands in STATA IC version 18.0 (21).

## 3 Results

Table 1 presents the demographic and health characteristics of the study population according to the presence of PUD. Significant differences were observed across several sociodemographic and health-related variables. The prevalence of peptic ulcer disease was higher among women than men (*p* = 0.004) and increased markedly with age (*p* < 0.001). Lower socioeconomic status, reflected by household income quintiles, was also associated with higher PUD prevalence (*p* = 0.005). A strong gradient was observed for self-perceived general health, with poorer health status associated with higher PUD prevalence (*p* < 0.001). Educational attainment showed an inverse association, with higher PUD prevalence among participants with lower education (*p* = 0.001).

**Table 1:** Demographic data and health status of the participants.

Variables	Category	Participants		*p*-Value
		without PUD, N (%)	with PUD, N (%)	
Sex	Male	2,322 (97.8)	51 (2.2)	**0**.**004**
	Female	2,701 (96.5)	98 (3.5)	
Age group	34 and younger	1,185 (99.4)	7 (0.6)	**<0**.**001**
	35–64 years old	2,408 (96.8)	80 (3.2)	
	65 and older	1,430 (95.8)	62 (4.2)	
Quintiles based on net equivalent household income	First (lowest)	1,043 (95.8)	46 (4.2)	**0**.**005**
	Second	1,077 (97.0)	35 (3.0)	
	Third	1,018 (97.2)	31 (2.9)	
	Fourth	1,225 (97.6)	28 (2.4)	
	Fifth (highest)	760 (98.7)	10 (1.3)	
Self-perceived general health status	Very good	852 (99.6)	3 (0.4)	**<0**.**001**
	Good	2,095 (99.1)	20 (0.9)	
	Satisfactory	1,525 (95.5)	71 (4.5)	
	Bad	426 (92.0)	37 (8.0)	
	Very bad	111 (85.1)	18 (13.1)	
Educational attainment	Primary	1,107 (95.8)	48 (4.2)	**0**.**001**
	Secondary	2,822 (97.1)	84 (2.9)	
	High	1,094 (98.5)	17 (1.5)	
Smoking status	Never smoked	2,741 (97.8)	62 (2.2)	**0**.**002**
	Former or active	2,441 (96.4)	85 (3.6)	
Alcohol consumption	Heavy alcohol user	246 (96.8)	8 (3.2)	**0**.**008**
	Moderate user	3,201 (97.6)	77 (2.4)	
	Non-alcohol user	1,536 (96.1)	63 (3.9)	
BMI	Normal (BMI < 25)	1,990 (97.5)	52 (2.6)	0.417
	Overweight (25 ≤ BMI < 30)	1,752 (96.7)	59 (3.3)	
	Obese (BMI > 30)	1,241 (97.0)	38 (3.0)	
Depression status (PHQ-8)	No depressive symptoms	4,680 (97.6)	114 (2.4)	**<0**.**001**
	Other depressive symptoms	168 (92.3)	14 (7.7)	
	Major depressive symptoms	91 (85.1)	16 (14.9)	
Cardiovascular diseases	No	4,652 (97.6)	114 (2.4)	**<0**.**001**
	Yes	371 (91.4)	35 (8.6)	
Rheumatic diseases	No	4,455 (97.7)	106 (2.3)	**<0**.**001**
	Yes	568 (92.9)	43 (7.1)	
Liver diseases	No	4,997 (97.2)	144 (2.8)	**<0**.**001**
	Yes	26 (83.9)	5 (16.1)	
Kidney diseases	No	4,897 (97.4)	133 (2.6)	**<0**.**001**
	Yes	126 (88.7)	16 (11.3)	

Bold values indicate statistical significance (*p* < 0.05) based on Chi-square test.

Lifestyle factors were also associated with PUD. PUD prevalence was higher among current or former smokers compared with never-smokers (*p* = 0.003), and non-alcohol users showed higher prevalence than moderate alcohol users (*p* = 0.008). Mental health status was strongly related to PUD, with markedly higher prevalence among participants with major depressive symptoms (*p* < 0.001). In addition, the presence of comorbid conditions, including cardiovascular, rheumatic, liver, and kidney diseases, was consistently associated with higher PUD prevalence (all *p* < 0.05).

Table 2 summarizes the associations between individual oral health indicators and the presence of PUD. Several indicators of poor oral health were significantly associated with PUD. Markers of active dental and periodontal disease, including decayed teeth, bleeding gums, and loose teeth, were associated with higher PUD prevalence (*p* = 0.045, *p* = 0.003, and *p* < 0.001, respectively), whereas the presence of filled teeth was associated with a lower prevalence of PUD (*p* = 0.002). Indicators reflecting cumulative oral disease burden, such as tooth extraction, dental restorations, and increasing numbers of missing teeth, were also significantly associated with higher PUD prevalence (all *p* < 0.001). Consistent with these findings, poorer self-perceived oral health showed a strong graded association with PUD prevalence (*p* < 0.001).

**Table 2:** Oral health status of the participants.

Variables	Category	Participants		*p*-Value*
		without PUD, N (%)	with PUD, N (%)	
Decayed tooth	No	3,613 (97.4)	96 (2.6)	**0**.**045**
	Yes	1,410 (96.4)	53 (3.6)	
Filled tooth	No	1,771 (96.2)	71 (3.8)	**0**.**002**
	Yes	3,252 (97.6)	78 (2.3)	
Bleeding gums when brushing	No	4,252 (97.4)	113 (2.6)	**0**.**003**
	Yes	771 (95.5)	36 (4.5)	
Loose tooth	No	4,638 (97.4)	123 (2.6)	**<0**.**001**
	Yes	385 (93.7)	26 (6.3)	
Tooth extraction due to decay/loosening	No	1,514 (98.5)	22 (1.5)	**<0**.**001**
	Yes	3,509 (96.5)	127 (3.5)	
Dental restoration (crown/bridge/denture/other)	No	2,653 (98.0)	54 (2.0)	**<0**.**001**
	Yes	2,370 (96.1)	95 (3.9)	
Missing teeth without dentures (excluding wisdom teeth)	No	2,174 (97.9)	45 (2.1)	**0**.**001**
	Yes	2,849 (96.5)	104 (3.5)	
Number of missing teeth	None	177 (98.9)	2 (1.1)	**<0**.**001**
	1–5 teeth	1,904 (97.8)	43 (2.2)	
	6–9 teeth	569 (97.1)	17 (2.9)	
	10–19 teeth	552 (95.2)	28 (4.8)	
	20 or more	875 (94.2)	54 (5.8)	
Self-perceived oral health status	Very good	527 (99.3)	4 (0.7)	**<0**.**001**
	Good	1,876 (98.3)	33 (1.7)	
	Satisfactory	1,609 (97.0)	49 (3.0)	
	Bad	746 (94.6)	43 (5.4)	
	Very bad	250 (93.0)	19 (7.0)	
Active dental and periodontal disease	No	3,098 (97.7)	74 (2.3)	**0**.**003**
	Yes	1,925 (96.2)	75 (3.8)	
History of dental and periodontal disease	No	1,151 (99.3)	8 (0.7)	**<0**.**001**
	Yes	3,872 (96.5)	141 (3.5)	

* Bold values indicate statistical significance (*p* < 0.05) based on Chi-square test.

Details of the EFA are provided in the Supplementary Material. The EFA of the seven oral health indicators confirmed data suitability (KMO = 0.54; Bartlett's test, *p* < 0.001) as shown in Supplementary Tables S1 and S2. Two factors were retained based on eigenvalues >1 and scree plot inspection, explaining 57.2% of variance. In the unrotated solution, Factor 1 explained 34.5%, and Factor 2 22.7% of variance; after varimax rotation, the explained variance was redistributed (Factor 1: 31.2%; Factor 2: 26.1%) (Supplementary Table S3). Factor 1, interpreted as active dental and periodontal disease, had strong loadings from decayed teeth (0.82), bleeding gums (0.67), and loose teeth (0.64). Factor 2, interpreted as a history of dental and periodontal disease, had strong loadings from tooth extraction (0.91) and dental restorations (0.74). Missing teeth without dentures loaded moderately on both factors (0.56 and 0.58) and was excluded due to redundancy with tooth extraction (tetrachoric correlation = 0.62). Absence of fillings had low loadings (0.33 and −0.03) and high uniqueness (0.89), indicating poor fit, and was excluded. Two subscales were created: active dental and periodontal disease (summing decayed teeth, bleeding gums, and loose teeth; range 0–3) and the history of dental and periodontal disease (summing tooth extraction and dental restorations; range 0–2) (Supplementary Table S4).

Based on the EFA results, 2000 responders had at least one indicator of active dental and periodontal disease, and 4,013 responders had at least one indicator of historical dental and periodontal damage. Participants with active dental and periodontal disease had a significantly higher prevalence of PUD compared to those without (3.8% vs. 2.3%, *p* = 0.003). Similarly, individuals with a history of dental and periodontal disease showed a significantly higher prevalence of PUD than those without (3.5% vs. 0.7%, *p* < 0.001). These findings suggest that both current and historical oral and periodontal diseases are associated with an increased likelihood of PUD (Table 2).

In the multivariable logistic regression model, several sociodemographic, behavioral, psychological, and health-related factors were significantly associated with the presence of PUD. Compared with participants aged 34 years or younger, both the 35–64-year-old group and the ≥65-year-old group showed higher odds of PUD. Female respondents had a significantly higher odds compared with males. Psychological status was also strongly associated with PUD. Individuals with other depressive symptoms had a more than two-fold higher odds, while those with major depressive symptoms had nearly a four-fold increased odds compared to participants without depressive symptoms.

Smoking was significantly related to PUD, with former or current smokers showing higher odds compared with never-smokers. In contrast, alcohol consumption, household income, and educational attainment were not significantly associated with PUD in the adjusted model. Among comorbid conditions, cardiovascular diseases, rheumatic diseases, liver diseases, and kidney diseases were all associated with higher odds of PUD.

Composite oral status derived from oral health-related variables was a significant determinant. Participants with active dental and periodontal disease had a 54% higher odds of PUD, while those with a history of dental and periodontal disease had more than twice the odds of PUD, compared with those without such conditions (Table 3).

**Table 3:** Logistic regression model.

Variable	Category/Level	OR	95% CI	*p*-Value*
Age group	34 and younger (ref)			
	35–64 years old	3.90	1.62–9.36	**0**.**002**
	65 and older	3.92	1.56–9.88	**0**.**004**
Sex	Male (ref)			
	Female	1.82	1.23–2.72	**0**.**003**
Depression status	No depressive symptoms (ref)			
	Other depressive symptoms	2.20	1.20–4.05	**0**.**011**
	Major depressive symptoms	3.83	2.04–7.18	**<0**.**001**
Smoking status	Never smoked (ref)			
	Former or active	1.71	1.18–2.46	**0**.**004**
Alcohol consumption	Heavy alcohol user (ref)			
	Moderate user	0.65	0.30–1.42	0.285
	Non-alcohol user	0.67	0.29–1.52	0.340
Quintiles based on net equivalent household income	First (lowest, ref)			
	Second	0.89	0.54–1.47	0.647
	Third	0.92	0.54–1.56	0.769
	Fourth	0.91	0.52–1.59	0.758
	Fifth (highest)	0.73	0.33–1.60	0.435
Educational attainment	Primary (ref)			
	Secondary	0.95	0.62–1.46	0.839
	High	0.75	0.38–1.45	0.405
Cardiovascular diseases	No (Ref)			
	Yes	2.03	1.28–3.21	**0**.**002**
Rheumatic diseases	No (Ref)			
	Yes	1.56	1.03–2.37	**0**.**034**
Liver diseases	No (Ref)			
	Yes	3.11	1.03–9.38	**0**.**043**
Kidney diseases	No (Ref)			
	Yes	2.11	1.14–3.90	**0**.**017**
Active dental and periodontal disease	No (Ref)			
	Yes	1.54	1.07–2.20	**0**.**017**
History of dental and periodontal disease	No (Ref)			
	Yes	2.17	1.01–4.67	**0**.**047**

* Bold values indicate statistical significance (*p* < 0.05). Odds ratios are adjusted for variables in the model. AUC = 0.707 (95% CI 0.668–0.746).

## 4 Discussion

In this nationally representative study of Hungarian adults, all oral health indicators showed consistent associations with PUD. After classifying these indicators into the composite oral health status of active dental and periodontal disease or the history of dental and periodontal disease based on the EFA results, active dental and periodontal disease was significantly associated with PUD after adjustment for age, sex, socioeconomic status, smoking, alcohol consumption, and comorbidities commonly linked to long-term NSAID use.

Our study results indicate that demographic factors such as older age and female sex are associated with the risk of PUD in the Hungarian population. Research demonstrates strong associations between age and PUD, with older age consistently identified as a significant risk factor. In Korean adults, age showed the highest association with PUD among all studied variables in both men and women (22). However, PUD affects all age groups, with its prevalence linked to lifestyle factors rather than age alone (23). The characteristics of PUD differ markedly by age and H.pylori status. Among patients with H. pylori-negative ulcers, 80.6% were aged 65 years or older, compared to only 33.7% of H. pylori-positive ulcer patients (24). For the variable of sex, the global epidemiological data have traditionally shown a male predominance in PUD prevalence and incidence; recent trends indicate narrowing sex differences and, in some age-specific cohorts, even higher rates in females. For instance, although males still have a slightly higher overall prevalence, the male-to-female ratio in PUD cases has declined to approximately 1:0.94 (25). In Denmark, a population-based cohort found that while lifetime ulcer prevalence was over twice as high in men, the five-year incidence showed no sex difference, suggesting convergence in risk between sexes (26). Further, in Korea, PUD bleeding incidence was higher in men until their 70s but rose in women over age 80, highlighting the role of age and possibly hormonal changes. These findings align with our results and underscore the evolving sex dynamics in PUD epidemiology.

The key modifiable lifestyle risk factors for PUD, included in our analysis, are cigarette smoking (27), heavy alcohol consumption (28), and chronic psychological stress (29). Cigarette smoking has been shown to be associated with biological processes such as increased gastric acid secretion, impaired mucosal defenses, and delayed ulcer healing, which may partly explain its observed association with PUD in epidemiological studies. It also enhances oxidative stress and inflammation, further aggravating mucosal damage (30). Alcohol consumption contributes to PUD by directly irritating the gastric mucosa, increasing gastric acid secretion, and impairing mucosal barrier function (31). Chronic psychological stress promotes PUD by activating the hypothalamic-pituitary-adrenal axis, which heightens gastric acid output and weakens mucosal defenses (32). These risk factors amplify H. pylori-induced inflammation and delay ulcer repair through increased oxidative stress (33). These mechanistic insights align with our findings, where smoking and depressive symptoms were independently associated with increased PUD risk, while alcohol consumption showed no significant association, consistent with variability in alcohol's role across populations.

In addition to lifestyle, the use of NSAIDs is one of the main risk factors for PUD. NSAIDs induce PUD through multiple mechanisms, primarily involving cyclooxygenase enzyme inhibition and direct mucosal injury, which impair gastrointestinal defense mechanisms (34). The pathogenesis includes epithelial cell membrane disruption and effects on the renin-angiotensin system, independent of cyclooxygenase enzyme pathways (35). Since our dataset does not include information on the use of certain medications, we included comorbidities that typically require long-term NSAID use for pain relief, anticoagulant, or anti-inflammatory purposes, such as cardiovascular diseases, rheumatic diseases, liver diseases, and kidney diseases, and the results showed significant associations. However, it should be noted that PUD is significantly associated with increased risks of cardiovascular disease and chronic kidney disease, driven by mechanisms beyond NSAID use, including H. pylori-induced inflammation and systemic oxidative stress. Studies show PUD patients face a 31% higher risk of ischemic stroke (36) and a 2–4 fold increased risk of myocardial infarction (37), while patients with chronic kidney disease have a 10–12 times higher PUD incidence (38), with H. pylori eradication reducing risks for both conditions. These findings suggest shared inflammatory and vascular pathways, highlighting the need for integrated management strategies.

Beyond demographic, lifestyle, and comorbidity factors, our findings underscore the independent association between poor oral health status and PUD. Several mechanisms may explain these findings. Evidence suggests a significant association between periodontal disease and the incidence of PUD. Periodontal disease is associated with systemic inflammatory processes and has been linked to gastrointestinal conditions, including PUD, in observational studies (14). Meta-analyses have reported improved treatment outcomes among PUD patients receiving periodontal therapy in addition to conventional ulcer treatment (39), supporting the hypothesis of a potential link between periodontal inflammation and ulcer disease, although causal pathways have not been definitively established. In a prospective cohort study involving male health professionals, researchers demonstrated that increased clinical attachment loss, a marker of periodontal disease, was associated with a higher incidence of PUD (40). These findings from longitudinal research suggest a possible temporal relationship; however, in the present cross-sectional study, the observed association may also reflect co-occurrence or reverse causality. Moreover, the systemic inflammatory burden caused by periodontitis can influence various metabolic and systemic diseases, potentially exacerbating conditions like PUD. A study finds a correlation between chronic gastritis and periodontal disease, reinforcing the hypothesis that periodontal health may affect gastric health (40). Potential biological pathways linking periodontal disease and peptic ulcer disease have been hypothesized to involve systemic inflammatory responses associated with pathogens present in dental biofilms; however, in the context of observational data, these mechanisms remain speculative and cannot be interpreted as causal. Several studies have reported the detection of *H. pylori* DNA in oral sites (41); however, the extent to which the oral cavity serves as a stable reservoir or contributes to gastric infection remains uncertain. These findings suggest a potential, but not definitively established, link between oral health, gastric inflammation, and ulcer formation.

In addition to the multiple regression results, chi-square test results show associations between all oral health indicators and PUD, each reflecting distinct aspects of oral disease burden. Decayed teeth may serve as protected niches for oral microorganisms, and some studies have reported the presence of H. pylori in dental caries and pulp tissue (42, 43); however, the clinical relevance of these findings for ulcer development remains debated. The absence of filled teeth, a proxy for untreated dental disease, was previously observed in ulcer patients who had fewer restorations and more oral debris than controls (44), suggesting that lack of treatment both reflects and reinforces poor oral environments conducive to microbial persistence. Bleeding gums, a hallmark of periodontal inflammation, have been consistently linked with PUD, with large-scale population studies in Asian countries showing significant associations between periodontitis and PUD (14, 15). Similarly, loose teeth, reflecting advanced periodontal destruction, may be associated with deeper periodontal pockets that coincide with higher systemic inflammatory burden (15). Indicators of cumulative oral disease, such as tooth extraction due to decay or loosening, likely capture long-term exposure to chronic infection and inflammation, may be associated with ulcer-related inflammatory processes (45). The role of dental restorations and prostheses remains less clear. Earlier studies suggested dentures were not a major reservoir for H. pylori (46), but more recent biofilm research indicates that prosthetic surfaces may provide niches that protect the organism from eradication therapy (47).

Although statistically significant, the observed associations, particularly for active dental and periodontal disease, were modest in magnitude, which is consistent with prior population-based studies. Previous cross-sectional studies from Korea and Japan have reported similarly modest effect sizes linking periodontal disease with PUD (14, 15), while stronger associations have occasionally been observed in longitudinal cohorts using clinically assessed periodontal measures (40). Differences in study design, reliance on self-reported oral health indicators, population age structure, healthcare access, and prevalence of Helicobacter pylori infection may partly explain variation in effect estimates across settings. Together, these findings suggest that poor oral health is likely one of several contributing factors associated with PUD rather than a dominant determinant. Given the cross-sectional nature of the data, alternative explanations must be considered. PUD and its treatment may adversely affect oral health through altered diet, medication use, or reduced healthcare utilization, raising the possibility of reverse causality.

The present study has several strengths. It is the first to examine the association between oral health and PUD in a nationally representative Hungarian population using standardized EHIS data, ensuring comparability with other European studies. Multiple oral health indicators were analyzed, and the construction of a composite oral health score allowed us to capture the cumulative burden of oral disease rather than relying on single markers. Furthermore, adjustment for a wide range of sociodemographic, lifestyle, mental health, and comorbidity variables reduced the risk of residual confounding.

However, certain limitations should be acknowledged. First, the cross-sectional design prevents causal inference, and the observed associations may reflect bidirectional or confounded relationships. Second, both peptic ulcer disease and oral health indicators were self-reported, introducing potential misclassification. Although PUD was assessed using a multi-step confirmation process, some misclassification cannot be excluded. Non-differential misclassification would likely bias associations toward the null, whereas differential misclassification related to health awareness or healthcare utilization could have affected the magnitude or direction of the observed associations. Third, information on *H. pylori* infection and medication use, particularly NSAIDs, which are established determinants of PUD, was unavailable. Although we attempted to partially account for medication-related confounding by including comorbidities commonly associated with long-term NSAID or antithrombotic therapy, the use of these comorbidities as proxy measures may have introduced residual confounding or overadjustment. Methodologically, the marginal overall KMO value suggests that the factor structure should be interpreted cautiously, and alternative approaches to summarizing oral health indicators may yield different results. In addition, depression and smoking may interact synergistically to influence ulcer risk through shared inflammatory, neuroendocrine, and behavioral pathways; while both factors were independently associated with PUD in our models, the present study was not designed to formally evaluate interaction effects. The EHIS questionnaire does not differentiate between gastric and duodenal ulcers, which may differ in etiology and risk factor profiles; therefore, the observed associations should be interpreted as relating to PUD overall rather than to specific ulcer subtypes. Finally, unmeasured factors such as diet, oral hygiene behaviors, and access to dental care may also influence the observed associations. Together, these limitations highlight the need for longitudinal, clinically verified studies with detailed medication and behavioral data to better clarify causal mechanisms.

## 5 Conclusion

Active dental and periodontal disease, as well as a history of these conditions, were independently associated with the presence of PUD after adjustment for major sociodemographic, lifestyle, and medical factors. Given the cross-sectional design, these findings should be interpreted as associative rather than causal. Nonetheless, they underscore the relevance of oral health status as a potential marker of gastrointestinal health and support the need for prospective studies to clarify temporal relationships and underlying mechanisms.

## Data Availability

The data analyzed in this study are subject to the following licenses/restrictions: The data presented in this study are available upon request from Hungarian Central Statistical Office who performed and supervised the data collection. Requests to access these datasets should be directed to Hungarian Central Statistical Office, www.ksh.hu/?lang=en (accessed on 1 April 2025). Requests to access these datasets should be directed to www.ksh.hu/?lang=en.
