# Supplementary material for: Association between oral health status and peptic ulcer disease: evidence from a nationally representative survey in Hungary
Source: Front Oral Health. 2026 Feb 18;7:1739255. doi: 10.3389/froh.2026.1739255 (PMC12956805; doi:10.3389/froh.2026.1739255)
Supplement: Supplementary file 1 [file Table1.docx]

**Supplementary Table S1.** Tetrachoric correlation matrix of oral health indicators

| Variable | Caries | Fillings | Bleeding gums | Loose teeth | Extraction | Prosthesis | Missing teeth |
| --- | --- | --- | --- | --- | --- | --- | --- |
| Caries | 1.00 | 0.23 | 0.35 | 0.41 | 0.22 | –0.31 | 0.41 |
| Fillings |  | 1.00 | 0.14 | –0.00 | 0.03 | –0.02 | 0.16 |
| Bleeding gums |  |  | 1.00 | 0.42 | 0.05 | –0.12 | 0.19 |
| Loose teeth |  |  |  | 1.00 | 0.34 | 0.01 | 0.35 |
| Extraction |  |  |  |  | 1.00 | 0.49 | 0.62 |
| Prosthesis |  |  |  |  |  | 1.00 | 0.01 |
| Missing teeth |  |  |  |  |  |  | 1.00 |

*Tetrachoric correlations were estimated due to binary indicators.*

**Supplementary Table S2.** Sampling adequacy and factorability diagnostics

| Indicator | KMO |
| --- | --- |
| Decayed teeth | 0.65 |
| Filled teeth | 0.41 |
| Bleeding gums | 0.68 |
| Loose teeth | 0.72 |
| Tooth extraction | 0.47 |
| Dental restorations | 0.33 |
| Missing teeth without dentures | 0.57 |
| Overall KMO | 0.54 |

*Bartlett’s test of sphericity: χ²(21) = 10,000, p < 0.001.*

**Supplementary Table S3.** Eigenvalues and variance explained (factor retention)

| Factor | Eigenvalue | % Variance | Cumulative % |
| --- | --- | --- | --- |
| 1 | 2.41 | 34.5 | 34.5 |
| 2 | 1.59 | 22.7 | 57.2 |
| 3 | 1.01 | 14.5 | 71.7 |
| 4 | 0.85 | 12.2 | 83.9 |

*Two factors were retained based on eigenvalues >1 and scree plot inspection.*

**Supplementary Table S4.** Rotated factor loadings and uniqueness

| Oral health indicator | Factor 1: Active disease | Factor 2: Disease history | Uniqueness |
| --- | --- | --- | --- |
| Decayed teeth | **0.82** | 0.02 | 0.32 |
| Bleeding gums | **0.67** | –0.05 | 0.55 |
| Loose teeth | **0.64** | 0.32 | 0.48 |
| Tooth extraction | 0.21 | **0.91** | 0.12 |
| Dental restorations/prosthesis | –0.43 | **0.74** | 0.27 |
| Missing teeth without dentures | 0.56 | 0.58 | 0.36 |
| Filled teeth (absence) | 0.33 | –0.03 | 0.89 |

*Loadings ≥0.40 were considered salient.*

*Missing teeth without dentures showed cross-loadings and was excluded due to redundancy with tooth extraction (tetrachoric r = 0.62).*

*Absence of fillings showed low loadings and high uniqueness and was excluded.*
